# Replication Cycle and Molecular Biology of the West Nile Virus

**DOI:** 10.3390/v6010013

**Published:** 2013-12-27

**Authors:** Margo A. Brinton

**Affiliations:** Department of Biology, Georgia State University, Atlanta, GA 30303, USA; E-Mail: mbrinton@gsu.edu; Tel.: +1-404-413-5388; Fax: +1-404-413-5301

**Keywords:** flavivirus replication, nonstructural proteins, nonstructural protein interactions, *cis*-acting RNA sequences, conserved RNA structures, host factors, RNA-protein interactions, host cell remodeling

## Abstract

West Nile virus (WNV) is a member of the genus *Flavivirus* in the family *Flaviviridae.* Flaviviruses replicate in the cytoplasm of infected cells and modify the host cell environment. Although much has been learned about virion structure and virion-endosomal membrane fusion, the cell receptor(s) used have not been definitively identified and little is known about the early stages of the virus replication cycle. Members of the genus *Flavivirus* differ from members of the two other genera of the family by the lack of a genomic internal ribosomal entry sequence and the creation of invaginations in the ER membrane rather than double-membrane vesicles that are used as the sites of exponential genome synthesis. The WNV genome 3' and 5' sequences that form the long distance RNA-RNA interaction required for minus strand initiation have been identified and contact sites on the 5' RNA stem loop for NS5 have been mapped. Structures obtained for many of the viral proteins have provided information relevant to their functions. Viral nonstructural protein interactions are complex and some may occur only in infected cells. Although interactions between many cellular proteins and virus components have been identified, the functions of most of these interactions have not been delineated.

## 1. Introduction

West Nile virus (WNV) is maintained in nature in a mosquito-bird transmission cycle and has recently become endemic in the Western hemisphere. All WNV isolates constitute a single serotype and the majority of WNV strains have been grouped into two lineages (1 and 2) based on signature amino acid substitutions and deletions in the envelope protein sequence [1]. Lineage 1 WNV strains are associated with outbreaks of human disease while lineage 2 viruses are primarily restricted to endemic enzootic infections in Africa [2,3]. Nevertheless, attenuated and virulent isolates from both lineages have been identified [4]. Both the recent extended geographic distribution and the increased frequency of human WNV outbreaks associated with a higher incidence of disease have enhanced the research effort focused on WNV infections. However, there are still many aspects of the virus replication cycle and virus-host interaction that are not yet well understood. 

WNV is a member of the family *Flaviviridae*, genus *Flavivirus*, and distantly related to the members of the two other genera in this family, *Pestivirus* and *Hepacivirus* [5]. All family members have single stranded, positive sense RNA genomes with a similar gene order and conserved nonstructural protein motifs. However, the genomes of the members of the different *Flaviviridae* genera differ in the *cis*-acting elements controlling viral RNA replication and translation as well as in some of the encoded genes. Translation of the pestivirus and hepacivirus polyproteins is initiated from an internal ribosome entry site (IRES) while the genomes of the members of the genus *Flavivirus* have a 5' Type I cap and no IRES. 

## 2. Virion Morphology, Attachment and Entry

Mature WN virions are small (~50 nm in diameter), spherical, and enveloped. The exodomains of envelop (E) protein dimers lie close to the outer surface of the virion membrane and are positioned head-to-tail. The membrane (M) protein contains two membrane spanning domains and a short ectodomain [6]. The E proteins encoded by most strains of WNV are glycosylated at a single *N*-linked glycosylation site (residue 154) [1,7]. The capsid (C) proteins, located inside virions, have no discernible nucleocapsid symmetry and no contacts between C proteins and either E or M on the inner side of the virion envelop have been observed [8]. Although nucleocapsid particles consisting of C protein and genome RNA are observed after removal of the virion envelop with nonionic detergents, capsid dimers can be dissociated from these structures by treatment with high salt [9]. C protein dimers have a very high net charge, with half of the basic residues located on one face and a conserved hydrophobic region that forms an apolar surface on the opposite face [10]. It is thought that the apolar surface of the C dimer interacts with the inner side of the virion envelop while the basic residue surface of the capsid dimer interacts with the genomic RNA. 

In culture, WNV replicates in various types of primary cells and immortalized cell lines from a wide variety of avian, mammalian, amphibian, and insect species suggesting that either highly conserved receptors and entry molecules are used or alternatively, that WNV may utilize different cellular proteins for these functions in different cell types and host species. In mice and humans, WNV tropism is more limited since infection targets monocytes, macrophages, dendritic cells, endothelial cells and neurons [11]. The cellular proteins functioning as co-receptors for virion attachment, entry and fusion have not yet been definitively characterized for WNV or any of the other flaviviruses. Various glycosaminoglycans can function as initial attachment contacts for flaviviruses [12]. Serial passage of WNV or Japanese encephalitis virus isolates in human adenocarcinoma (SW13) cells produced virus with a small plaque phenotype, an increased affinity for heparin-sepharose and attenuated neuroinvasiveness in mouse models of flavivirus encephalitis [13]. These results were interpreted to indicate that the passage variants had an altered interaction with surface glycosaminoglycans involved in attachment or entry. Even though the dendritic cell-specific lectins, DC-SIGNR and DC-SIGN, can both bind mannose-rich glycans, only DC-SIGNR could efficiently promote a WNV infection [14]. Glycosylation of either prM or E is sufficient for WN virion interaction with DC-SIGNR. However, WNV efficiently infects many types of cells that do not express DC-SIGNR and can also bind to red blood cells [15]. Phosphatidylserine binding proteins of the TIM family were recently reported to facilitate infection by a number of different types of enveloped viruses, including WNV, through interaction with virion-associated phosphatidylserine [16]. Direct interaction of WNV with cell surface TIM proteins as well as indirect interactions with cellular TAM proteins may explain the broader tropism observed for WNV in cultured cells. 

Cellular α_v_β_3_ integrin and laminin-binding protein were reported to function as WNV receptors [17,18] but the use of α_v_β_3_ integrin as a WNV receptor was not consistently supported by data from subsequent studies. Even though several flaviviruses including WNV contain an integrin recognition RGD/RGE motif within the domain III region of their E proteins, RGD peptides did not competitively inhibit WNV entry [17]. However, the WNV E protein domain III was subsequently reported to interact with α_v_β_3_ integrin in both co-immunoprecipatation and receptor competition assays [19]. The binding efficiency of multiple strains of WNV to cells was not affected by the lack of expression of the integrin subunits αv, β1 or β2 but reconstitution of the expression of β1 or β2 enhanced virus yield [20]. Activation of β1 and β2 integrins by protein disulfide isomerase was reported to facilitate dengue entry [21]. However, WNV internalization was shown not to require either α_v_β_3_ integrin or focal adhesion kinase but instead to be dependent on the lipid raft pathway [22]. siRNA screens identified G-coupled receptor kinase (GRK) family proteins as important host factors for WNV, yellow fever virus and dengue virus, [23,24,25]. Confirmation studies with yellow fever virus and HCV indicated that GRK2 enhances both virus entry and viral RNA synthesis [26]. Although knock down of the ubiquitin ligase CBLL1 was reported to inhibit internalization of WNV [24], a subsequent study did not confirm a requirement for CBLL1 during WNV entry [27]. Mosquito C-type lectin, mosGCTL-1, was shown to bind to WNV in a calcium dependent manner and interaction of the mosGCTL-1-WNV complex with the mosquito CD45 homolog, mosPTP-1, was reported to enable virus attachment and enhance entry into mosquito cells [28]. 

WN virions enter both mammalian and mosquito cells via receptor-mediated endocytosis of clathrin-coated pits [17,29]. Rab 5 was reported to be required for WNV and dengue entry [30]. After internalization, dengue virus particles were shown to be delivered to early or intermediate endosomes which mature to late endosomes [31]. As the environment of the endosome acidifies, the virion E proteins are triggered to irreversibly associate into extended fusion trimers that mediate viral and endosomal membrane fusion [32,33]. The capsid dimers would be expected to remain associated with the inner surface of the virion membrane which, after viral-endosomal membrane fusion, is located on the outer surface of the endosome. The entering viral genome may remain associated with the capsid dimers. This association may help to protect the incoming viral genome from cellular nucleases and RNA sensors as well as provide a “scaffold” on the outer surface of endosomes for the genome as it switches back and forth between initial rounds of translation and replication. In support of this hypothesis, flavivirus C proteins were reported to be able to function as RNA chaperones [34,35]. 

## 3. Early Effects of Infection on Cells

Rapid early and sustained Ca^2+^ influx into MEFs in response to WNV infection was detected with Ca^2+^ entering during virion endocytosis as well as through calcium channels [36]. Activation of the FAK, ERK1/2, and Akt kinases at early times after WNV infection was dependent on Ca^2+^ influx, but not on α_v_β_3_ integrins, and this activation was sustained throughout the viral replication cycle. In contrast, UV-inactivated WNV induced only transient early kinase activation. Treatments that reduced Ca^2+^ influx at early times of infection decreased viral yield suggesting that Ca^2+^ influx enhances early events needed to ensure efficient WNV replication. Increased caspase 3 cleavage at both early (transient) and late times of infection correlated with decreased FAK and ERK1/2 activation, indicating that activation of these kinase pathways extends the survival of flavivirus-infected cells. Expression of WNV capsid alone in A549 cells was also reported to enhance cell survival through activation of AKT [37].

## 4. Viral Genome RNA

**Figure 1:**
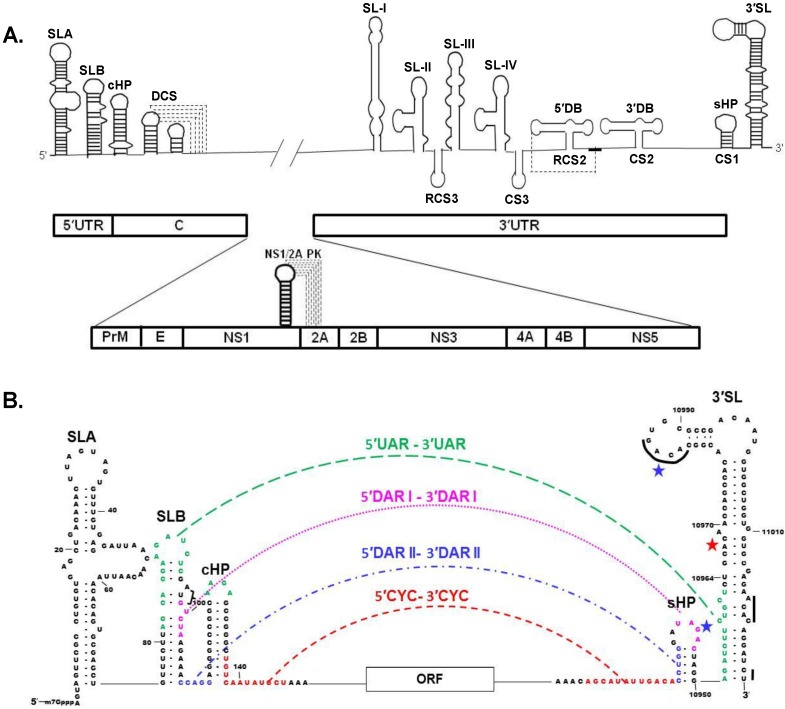
Schematic representations of the West Nile Virus (WNV) genomic RNA. (**A**) Conserved stem loop (SL) RNA structures located in the 3' and 5' untranslated regions (UTRs) as well as the capsid coding region are shown in the top panel. Pseudoknot interactions are indicated by dotted lines. The positions of the individual virus protein coding regions in the polyprotein ORF are shown in the bottom panel. Viral protein order from the N terminus of the polyprotein: capsid (C), pre-membrane (prM), envelop (E), and the nonstructural proteins, NS1 through NS5. A pseudoknot involved in a -1 translational frameshift that is required for the production of NS1' is also depicted in the lower panel; (**B**) Terminal region genome sequences that are involved in 3'–5' long distance RNA-RNA interactions. The interacting 5' upstream of the AUG (UAR) sequence and its 3' complement are indicated in green, the 5' downstream of the AUG 1 (DAR1) sequences in pink, the 5' downstream of the AUG 2 (DAR2) sequences in blue and the cyclization (CYC) sequences in orange. The black lines on the 3' SL indicate conserved sequences. The major binding site for eEF1A is indicated with a red star and the minor eEF1A binding sites are indicated by blue stars. Bracket, AUG of ORF.

The WNV genome is a single-stranded, positive sense RNA of ~11,000 nt [3]. The genome functions as the only viral mRNA and also as the template for synthesis of the complementary minus strand RNA. The 5' non-coding region (NCR) of the WNV genome is 96 nts in length, while the length of the 3' NCR varies from 337 to 649 nts. The variable region of the 3' NCR is located just 3' of the coding region stop codon [38]. The 3' end of the genome RNA does not contain a poly A tract but instead terminates with a conserved CU_OH_ (Figure 1B) [39,40,41]. The 3' and 5' terminal sequences of the genome fold into RNA secondary structures that are conserved among divergent flaviviruses even though the majority of the nts composing these structures are not conserved (Figure 1A). [40,42,43]. Deletion of either the 3' or 5' terminal genomic stem loop (SL) sequences is lethal for flavivirus infectious clones [44,45,46]. The 3' terminal RNA structures were initially analyzed by structure probing [40] and more recently by NMR spectrometry [47] and also by selective 2'-hydrozyl acylation analyzed by primer extension (SHAPE) [48]. Short conserved sequences within the 3' terminal SL structure of flavivirus genomic RNA include, the terminal 5'-CU-3', a 5'-ACAC-3' sequence near the 3' terminus and a 5'-ACAG-3' in the top loop of the 3' terminal SL (indicated by black lines, Figure 1B) [40]. Mutation of individual nts in the top loop of a WNV infectious clone indicated that the majority were *cis* acting and that the three underlined nts (5'-ACAGUGC-3') were required for virus viability [49,50]. A small conserved SL (sHP, also named SSL) is located adjacent to the 3' terminal SL (Figure 1A,B). Deletion of the sHP in a WNV infectious clone or the introduction of mutations that disrupted the sHP stem in a dengue infectious clone was lethal [47,51]. The existence of a previously predicted pseudoknot between 4 loop nts of the WNV sHP and nts on the 5' side of the 3' terminal SL [52] was not confirmed by a recent NMR structural analysis [47]. Interestingly, it was reported that nt substitutions in the loop and stem of a dengue virus sHP had a greater negative effect on virus replication in C6/36 mosquito cells than in mammalian cells [51].

A number of additional RNA structures in the 3' NCR have been identified. The direct repeats (CS2 and RCS2) form “dumbbell-like” secondary structures (3' DB and 5' DB, Figure 1A) and it was suggested that these repeats are evolutionary remnants of an ancient sequence consisting of 6 repeats [53,54]. Mutational analyses of these sequences suggested that they are functionally important for both RNA replication and translation [55]. The formation of pseudoknots between 5 nts in the terminal loop sequence (TL) of each of the dumbbell structures and a complementary sequence located 3' of the 3' dumbbell structure was previously predicted [54]. A recent SHAPE analysis provided support for the existence of a pseudoknot interaction for only the TL of the 5' DB (Figure 1A) [48]. Four additional SLs, named SL-I through IV (Figure 1A), are located 5' of the dumbbell structures. SL-II and SL-IV were predicted to be involved in pseudoknot interactions and SL-II was reported to stall the cellular 5'–3' exoribonuclease XRN1, resulting in incomplete digestion of the viral genome RNA and generation of the 3' subgenomic flavivirus RNA (sfRNA) [56].

**Figure 2:**
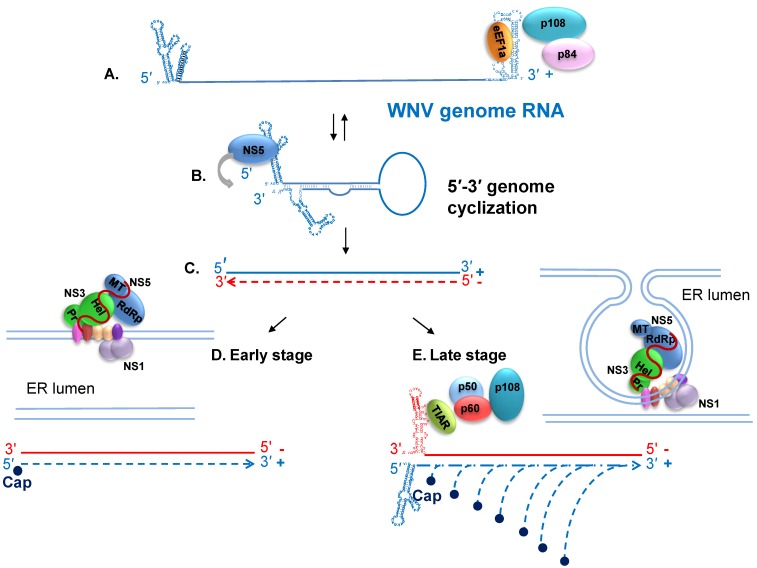
Diagrams of WNV RNA replication at different phases of the viral replication cycle. (**A**) The linear form of the plus strand RNA genome is utilized as a template for translation; (**B**) The genome switches back and forth between linear and cyclized forms. The interaction of the cell protein eEF1A with three sites on the 3' stem loop (SL) RNA may facilitate the opening of 3' region basepairs prior to formation of the 3'-5' RNA-RNA interaction that cyclizes the genome. The cyclized form of the genome functions as the template for minus strand RNA synthesis. NS5 recruitment to the 3' end of the minus strand is facilitated by its interaction with the 5' SLA; (**C**) A single minus stand is copied from the genome; (**D**) Release of the 3' end of the minus strand from the template-product duplex by an unknown mechanism leads to initiation of the synthesis of a plus strand (genome) RNA. The viral replication complexes are associated with ER membranes at early stages of replication but ER invaginations are not yet observed and reinitiation of plus strand RNA synthesis is inefficient; (**E**) At later stages of the replication cycle, viral nonstructural proteins have induced the formation of invaginations in the ER membrane that contain viral replication complexes, cell proteins and minus strand templates. Late stage reinitiation of plus strand synthesis is very efficient. The complex formed by TIAR and other cell proteins and the 3' terminal SL of the minus strand is thought to enhance recruitment and possibly also the positioning of NS5 for plus strand reinitiation. Viral nonstructural protein: NS1 (medium blue), NS2A (dark red), NS2B (pink), NS3 (green), NS4A dimer (peach), and NS4B (purple).

Conserved RNA structures are also located at the 5' end of the genome [42]. The 5' terminal nts form SLA, the polyprotein AUG is located in the adjacent SLB which is followed by a stable hairpin (cHP) (Figure 1A,B). The cHP was reported to be involved in the selection of the codon used for translation initiation [57]. The 3' terminal nts of the minus strand RNA form a conserved SL structure and although the nts composing this structure are complementary to the 5' nts of the genome, the SL structures differ due to the formation of G-U base pairs (Figure 2E) [40,58]. The 5' end of the WNV genome contains a type 1 cap structure (m^7^GpppAmp) that is added by NS5 during genome transcription. The *N*-terminal region of NS5 contains guanylyltranferase as well as both N7 and 2'-*O S*-adenosyl methionine methyltransferase (MTase) activities [59,60,61]. Repositioning of the 5' end of a nascent genomic RNA on the MTase is thought to be required for each sequential modification in the capping process to occur [62,63]. The NS5 MTase contact sites on the WNV 5' RNA required for each of the sequential methyltransferase reactions have been mapped [64]. 2'-*O* methylation of the flavivirus cap allows the viral genome to escape restriction by the innate immune response IFIT family members [65].

### Long Distance 3′-5′ RNA-RNA Interactions

A single copy of a sequence designated conserved sequence 1 (CS1) is located upstream of the 3′ terminal SL and partially overlaps the sHP in mosquito-borne flavivirus genomes (Figure 1A). A highly conserved 8 nt sequence, designated the 3' cyclization sequence (3' CYC), is located within the 5' portion of CS1 and is an exact complement of the 8 nt 5' CYC sequence located in the capsid coding region near the 5' end of the genome (Figure 1B) [66]. Cyclization of the genome, due to the 3'–5' long distance RNA-RNA interactions, was shown to be required for minus strand RNA initiation but not for translation. [67,68,69,70]. Although an initial study suggested that only base pairing between the 3' and 5' CYC sequences and not the sequences themselves was required for genome cyclization [69], a subsequent study showed that changing the sequences of some 3'–5' CYC basepairs reduced replication efficiency [71]. Although the replication of a flavivirus replicon was completely inhibited by single or multiple mismatching CYC nt substitutions, two or three mismatching CYC substitutions made in a WNV infectious clone caused reduced replication efficiency and revertants with increased replication efficiency were rapidly generated in BHK cells [71]. Several different mutant genomes with three adjacent mismatching CYC substitutions were rescued by the same spontaneous single second site mutation that created an additional basepair (nts 147–10,913) on the internal genomic side of the 5'–3' CYC. However, mutations that disrupted five adjacent CYC basepairs were lethal in the context of a full length infectious clone [71]. Additional terminal genomic sequences were shown to also be involved in the long distance 5'–3' RNA-RNA interactions. The 5' upstream AUG region (UAR) interacts with a complementary 3' UAR sequence located downstream of the 3' CYC (Figure 1B) [72,73] and this interaction was shown to be functionally important [74]. Also, two (WNV) or one (dengue 2) 5' downstream of the AUG codon region (DAR) sequence(s) [75,76] interact with the complementary 3' DAR sequence(s) located between the 3' UAR and 3' CYC sequences (Figure 1B). Interestingly, deletion of the entire 3' CYC in a WNV infectious clone severely reduced virus replication but revertant viruses were recovered with mutations in the UAR and DAR regions that partially compensated for the loss of the 5'–3' CYC interaction [77].

Interaction between 5' and 3' flavivirus terminal genome sequences was demonstrated by atomic force microscopy [72], structure probing (Figure 1C) [75,78], and electrophoretic mobility shift assays [72,73]. RNA fragments with mutations that disrupted CYC basepairing were not able to form the 5'–3' CYC or UAR interactions *in vitro*, while mismatching UAR mutations disrupted the UAR interaction but had no effect on the CYC interaction [78]. It has been proposed that the flavivirus genomic 5'–3' RNA-RNA interaction initiates between the 5' CS and 3' CS, that the interaction of the 5'–3' DAR sequences then extends the initial long distance interaction, and that this assists the long distance interaction of the UAR elements which unwinds the bottom of the terminal 3' SL [79]. Data from several studies indicated that either sequence or a pseudoknot (DCS) located downstream of the 5' CYC could also facilitate genome cyclization (Figure 1A) [71,80,81]. The cellular protein La [82], the viral capsid protein [83] and the viral NS3 protein [84] have been reported to be able to facilitate flavivirus genome 3'–5' RNA-RNA annealing *in vitro*. 

The 3' sHP and the 5' cHP are present only in the context of the “linear” form of the genome since some of the nts in the stem regions of these structures form long distance basepairs in the cyclized form of the genome (Figure 1B,C). Mutations in these regions that shifted the balance between the two forms of the genome and negatively affected virus replication spontaneously reverted due to second site mutations that restored the balance between the linear and cyclized forms [85,86,87]. These findings suggest that the correct balance between linear and circular forms of the genome is important to ensure efficient viral replication. The interaction of NS5 with sites on the 5' terminal SLA when the 3' end of the genome is in close proximity in the context of the 3'–5' long distance RNA-RNA interaction has been reported to facilitate the interaction of NS5 with the 3' nts leading to the initiation of viral minus strand RNA synthesis (Figure 2B) [75,88].

## 5. Viral Polyprotein

A single open reading frame (ORF) of 10,301 nt in most WNV isolates encodes a polyprotein that is co- and post-translationally processed by the viral serine protease complex (NS2B-NS3) and various cellular proteases into 10 mature viral proteins [89,90]. The three viral structural proteins, capsid (C), membrane (prM/M) and envelop (E), are encoded within the 5' portion of the ORF, while the 7 nonstructural proteins (NS1, NS2A, NS2B, NS3, NS4A, NS4B, and NS5) are encoded within the 3' portion (Figure 1A) [39]. The viral polyprotein contains multiple transmembrane domains that determine whether individual mature viral proteins are located on the cytoplasmic or luminal side of the endoplasmic reticular (ER) membrane after cleavage from the polyprotein [91]. The C, NS3 and NS5 proteins are located on the cytoplasmic side while the PrM, E, and NS1 proteins are in the lumen and, with the exception of short regions between transmembrane domains, the NS2A, NS2B, NS4A and NS4B proteins are located within the ER membrane bilayer [91]. 

## 6. Viral Nonstructural Proteins

Although the functions of the WNV nonstructural proteins have not yet been completely characterized, all seven are directly or indirectly involved in viral RNA synthesis and additional functions for some of these proteins have been identified. Little is known about the interactions between the viral nonstructural proteins or between viral nonstructural proteins and cell proteins that are required for remodeling the cell environment and for appropriately regulating active viral RNA replication complexes at different phases of the virus life cycle. 

### 6.1. Four Nonstructural Membrane Proteins

NS2A, NS2B, NS4A and NS4B are small, hydrophobic proteins that do not have conserved motifs characteristic of known enzymes. The structures and functions of these four proteins have not yet been well characterized. Each of these proteins has two or three membrane-spanning regions and facilitates the assembly and/or anchoring of viral replication complexes on the ER membrane [92,93,94,95]. Neither NS2A, NS2B, NS4A, nor NS4B could be complemented *in trans* in a Kunjin replicon system [96]. However, partial *trans* complementation of a dengue virus with a lethal NS4B point mutation was reported [97]. NS2A has been reported to also be involved in virion assembly and IFN-independent apoptotic cell death [92,98,99,100]. Recently, an ER membrane topology model for dengue virus NS2A was reported [101]. The *N*-terminal amino acids are located in the ER lumen while the *C*-terminal tail is in the cytosol. The first of the five transmembrane regions located in the middle part of NS2A consists of two helices separated by the “helix-breaker” amino acids P85 and R84. Mutation of each of these residues in both a replicon and an infectious clone showed that R84 but not P85 was functionally important. Interestingly, an R48E mutation attenuated both viral RNA replication and virion production while an R84A mutation had no effect on viral RNA synthesis but inhibited the production of infectious virions. NS2B interacts with the NS3 *C*-terminal protease domain and functions as a protease co-factor [102]. The *C*-terminal region of NS4A can be cleaved by cell signalase generating the 2K fragment. Overexpression of Kunjin NS4A-2K or dengue NS4A in mammalian cells induced cytoplamic membrane rearrangements that were similar to those observed in infected cells [92,95,103]. *In silico* analyses of the membrane topology of assembled NS4A structural domains supported the capacity of NS4A to induce membrane curvature [104]. An *N*-terminal amphipathic helix in the dengue virus NS4A was reported to facilitate homo-oligomerization and mutations that disrupted this helix completely inhibited virus replication [105]. Induction of cytoplasmic membrane proliferation was observed when Kunjin NS4B was expressed in mammalian cells [106]. Expression of WNV NY99 and Kunjin NS2A, NS2B, NS4A or NS4B was reported to block Type 1 IFN signaling [107,108,109]. WNV with the NS4B mutations P38G/T116I/N480H had a temperature sensitive and small plaque phenotype and showed a >10^7^ attenuation of neuroinvasiveness and a lower titer viremia in mice. Five different spontaneous compensatory mutations in NS4B reduced temperature sensitivity and increased virulence [110,111]. An alternative reading frame in the NS4B region of linage 1 WNV genomes that could be translated after a-1 frame shift was reported [112]. A subsequent study showed that the predicted N-NS4B/WARF4 protein containing the *N*-terminal region of NS4B and a unique *C*-terminus was produced by lineage 1 WNV infected Vero cells and also that antibodies to N-NS4B/WARF4 were produced by some but not all of the WNV-infected horses tested [113]. The function(s) of this protein is not currently known. 

### 6.2. Nonstructural Protein 1

NS1 is a glycoprotein with three conserved *N*-linked glycosylation sites and multiple conserved cysteines that form disulfide bonds essential for virus viability [114,115,116]. The location of NS1 on the lumenal side of the ER membrane after it is processed from the polyprotein is determined by the *C*-terminal E protein signal sequence [117]. NS1 monomers are soluble and hydrophilic while NS1 homodimers associate with membranes [118,119]. NS1 is also secreted by mammalian cells as a soluble hexamer [120,121,122,123,124,125]. Although NS1 is located on the luminal side of the ER membrane, it can colocalize with viral replication complexes located on the other side of the ER membrane in infected cells (Figure 2) [126,127]. Mutation of NS1 *N*-linked glycosylation sites in the yellow fever virus genome dramatically reduced viral RNA replication levels [128]. However, another study observed near wildtype viral replication levels in cell culture with the WNV NS1 (130-132QQA/175A/207A) mutant but this mutant was attenuated for both neuroinvasiveness and neurovirulence in mice [116]. A yellow fever virus with a temperature sensitive mutation in NS1 was defective in viral RNA synthesis at the non-permissive temperature [129]. A yellow fever virus genome with a large in-frame deletion in NS1 was not able to replicate viral RNA unless NS1 was supplied *in trans* suggesting that NS1 is required for the initiation of RNA synthesis and may have an essential function during early minus strand synthesis [89]. 

In WNV infected Vero cells, NS1 secretion begins between 16 and 24 h after infection while the release of virus particles begins between 8 and 16 h [130]. Secreted soluble dengue NS1 was reported to bind to glycosaminoglycans on the surface of infected cells in a cell type specific manner [131]. Anti-NS1 antibodies are elicited in infected hosts and facilitate elimination of infected cells [132]. Although an initial report suggested that extracellular WNV NS1 inhibited TLR3 signal transduction [133], this was not confirmed by a subsequent study [134]. Both secreted soluble and cell surface NS1 counteract the protective effects of the complement system by binding and recruiting complement regulatory factor H which decreases complement activation in solution and reduces cell surface deposition of C3 fragments and C5b-9 membrane attack complexes [135]. NS1 also interacts with C4, which reduces the deposition of C4b and the activity of C3 convertase [136]. The interaction of NS1 with both C1 and C4 promotes degradation of C4 to C4b. This mechanism protects virions in solution from complement-dependent neutralization. Glycosylation of NS1 at the 130 position stabilizes the secreted hexamer and enhances NS1 binding to the complement components C1s and C4 while glycosylation at the 207 position facilitates both the secretion and stability of the hexamer [137]. A proline to leucine substitution at position 250 in Kunjin (WNV) NS1 completely inhibited NS1 dimer formation, but not secretion, and reduced virus production in Vero cells and virus infectivity in weanling mice [138]. A mutant WNV NY99 with asparagine to alanine substitutions at the three *N*-linked glycosylation sites in NS1 as well as in the E protein glycosylation site (N154S) replicated only slightly less efficiently than the parental virus in cell culture but showed a 200,000 fold reduction in neuroinvasiveness [139,140]. A reversion at position 130 was detected in virus isolated from mice that succumbed to infection with this mutant virus suggesting that this was a critical position. When the *N*-linked glycosylation site asparagines were substituted with either serine or glutamine instead of alanine and all three residues of the 130 *N*-linked glycosylation site were substituted in the mutant NS1 (130-132QQA/175A/207A), complete attenuation of neuroinvasiveness was observed [116]. 

Multiple bands of NS1 are detected in infected cells by Western blotting due to variable glycosylation (glycosylation can add up to 6 kDa to the mass of NS1), to the formation of multiple disulfide bonds and to the existence of two different forms of NS1 [125]. The longer form named NS1' has the *N*-terminal NS1 sequence and an additional unique *C*-terminal sequence from a different overlapping ORF that extends into the NS2A region. NS1' was subsequently shown to be the product of a-1 ribosomal frame shift mediated by a slippery sequence followed by a pseudoknot (Figure 1A) [141,142]. Only the genomes of members of the Japanese encephalitis serogroup contain this pseudoknot and produce NS1'. NS1' was shown to associate with viral replication complex proteins on cytoplasmic membranes [143] and to be able to perform the functions of NS1 in viral RNA replication in trans-complementation experiments [144]. Both Kunjin and Japanese encephalitis viruses with mutations that ablated the production of NS1' had reduced neuroinvasiveness phenotypes but the mechanism involved is not yet known [142,145]. The *C*-terminal region of NS1' was reported to contain a site that can be cleaved by caspases during apoptosis [146]. A WNV replicon with a mutation in NS2A that eliminated the production of NS1' packaged virus particles more efficiently suggesting the possibility that NS1/NS1' may regulate virion assembly [147]. 

### 6.3. Nonstructural Protein 3

The *N*-terminal 175 residues of NS3 comprise a serine protease that is active only when complexed with NS2B [148,149,150]. The NS3-NS2B protease complex cleaves the viral polyprotein at multiple sites consisting of two basic amino acids followed by a short side chain amino acid [151]. Membrane association of the NS3-NS2B complex is required for efficient polyprotein processing. Although it was previously thought that NS3 associated with the ER membrane only indirectly through its interaction with the membrane spanning NS2B protein, a recent study of the interaction of NS3 with micelles found that the NS3 amino acids 31 to 33 can interact with membrane and it was proposed that this region may anchor and stabilize the *N*-terminal NS3 protease domain on membranes [152]. However, association of NS3 with the ER membrane was not observed when NS3 was expressed alone in mammalian cells suggesting that the direct interaction of NS3 with membrane is weak and not sufficient to tether NS3 to membranes in the absence of NS2B [153]. 

Initial crystal structures obtained for the NS3 protease fused to the co-factor NS2B and in some cases, also to an inhibitor, indicated the existence of two conformations of the catalytic histidine and suggested that the ligand plays a role in stabilizing the catalytically competent His conformation [154,155]. In the closed conformation, which was only found in the presence of a ligand/inhibitor, NS2B wraps around NS3 and completes the active site by contributing a β-fold to the chymotrypsin-like fold and also provides a negatively charged surface at the S2 site [155]. In the open inactive configuration, the NS2B chain changes direction shortly after entering the *C*-terminal lobe of NS3. A recent NMR analysis of an unlinked NS2B-NS3pro complex found that this complex predominantly assumes the closed conformation in solution [156]. Crystal structures of NS2B fused to full length NS3 indicated that the *N*-terminal protease and *C*-terminal NTPase/helicase domains of NS3 are segregated globular domains that are expected to have little structural influence on each other [157,158]. The different orientations of the protease and NTPase/helicase domains in the structures obtained by these two studies indicate that the two domains are joined by a flexible interdomain linker. 

RNA-stimulated nucleoside triphosphatase (NTPase) [41], ATPase/helicase [149,159], and 5'-triphosphatase (RTPase) [160] activities have been demonstrated for the *C*-terminal domain of NS3. Crystal structures indicate that the NTPase overlaps the first two helicase domains and that the third helicase domain interacts with viral RNA and other proteins [161,162]. The *C*-terminal RTPase utilizes the Walker B motif of the helicase-NTPase catalytic center for phosphodiester bond hydrolysis [163]. The RTPase dephosphorylates the 5' end of nascent viral RNAs prior to cap addition. The divalent cation dependent RTPase activity is not specific for the viral 5' RNA and can work on any protruding 5' terminus [164]. The flavivirus helicase preferentially binds to RNA over DNA but in a sequence independent manner and can unwind a 3' tailed duplex with an RNA but not a DNA loading strand. The binding of the NS3 helicase to single stranded RNA at the end of a duplex triggers a conformational change that results in a catalytically active enzyme that unwinds double stranded RNA driven by RNA-stimulated nucleoside triphosphate hydrolysis [165,166]. The NS3 helicase is poorly processive and NS4A was reported to act as a cofactor for the NS3 helicase helping to sustain its activity when ATP levels are low [167]. In addition to its NTPase and double stranded RNA unwinding activities, dengue virus NS3 was recently reported to be able to enhance annealing of complementary RNA sequences in an ATP-independent manner and it was proposed that the ATP concentration regulates the steady state between the unwinding and annealing functions of NS3 [84]. 

### 6.4. Nonstructural Protein 5

NS5 is located at the *C*-terminus of the viral polyprotein (Figure 1A) and is the largest and most conserved of the flavivirus proteins. The *N*-terminal region of NS5 contains an *S*-adenosyl methionine methyltransferase (MTase) domain that has both N7 and 2'-*O* MTase activities and also acts as a guanylyltransferase [59,61]. Flavivirus MTases have an active center that sequentially transfers a methyl group onto two acceptor positions that differ from each other chemically and conformationally with the N7 methyl transfer preceding the 2'-*O* methyl transfer. N7-methylation of the genome RNA requires specific unpaired 5' nts on the genome RNA at the second and third positions followed by the 5' terminal SL, while 2'-*O*-methylation requires specific nts at the first and third positions followed by a minimum of 20 nts [75,168]. Removal of one or both 5' phosphates on the RNA reduces NS5 MTase binding [169]. A co-crystal structure of the dengue MTase bound to a 5' capped octomeric RNA was hypothesized to represent the conformation present after guanylylation of the genome RNA [170]. 

The *C*-terminal portion of NS5 contains conserved sequence motifs characteristic of all RNA-dependent-RNA polymerases (RdRps) [171,172]. Two adjacent nuclear localization signals in dengue NS5, βNLS (aa 320 to 368) and α/βNLS (aa 369 to 405), were reported to bind to importin β as well as to a heterodimer of importin α/β with high affinity *in vitro* [173]. Although nuclear as well as cytoplasmic localization of dengue NS5 have been reported, the αβNLS region was subsequently shown to be a functional element of the RdRp domain required for polymerase activity [173,174,175], and nuclear localization of NS5 was shown to have little effect on virus replication [175,176]. 

Crystal structures of the NS5 RdRps of WNV and dengue virus [177,178] revealed canonical right hand RdRp structures consisting of finger, palm and thumb domains with a priming loop that differed in its fold from that of the hepatitis C RdRp. Trp 800, which is conserved among the members of the genus *Flavivirus*, is the critical priming loop residue required for *de novo* initiation and provides the initiation platform due to stacking of its aromatic base against the priming nt [177]. Structural analysis of the flavivirus RdRp domains revealed two conserved cavities in thumb subdomains and mutation of amino acids in these cavities in either a dengue infectious clone or a recombinant protein identified one residue (Lys765) in cavity A that was critical for virus replication but did not affect RdRp activity, three residues (Lys330, Trp859 and Ile863) that are required for initiation of RNA synthesis and one (Lys330) that interacts with NS3 in cavity B [179]. Another study showed that dengue NS5 with substitutions K456A and K457A in the F1 motif was able to efficiently synthesize product from a non-specific poly (rC) template but was unable to initiate synthesis from a flavivirus SLA promoter even though neither the efficiency of the NS5-SLA interaction nor of product elongation from a primer was affected [180]. 

The recent successful crystallization of the full length Japanese encephalitis virus NS5 revealed for the first time intra-molecular interactions between the MTase and RdRp domains [181]. The *C*-terminus of the MTase is connected to the RdRp by a variable 10 amino acid linker. In a previous study, substitution of amino acids 46, 47 and 49 in the αA3 motif of the MTase in a dengue infectious clone severely reduced virus replication but this mutant was rescued by a spontaneous second site mutation at amino acid 512 in the palm of the RdRP suggesting intra-domain interaction between these residues [177]. However, although co-expressed WNV MTase and RdRp domains could be co-immunoprecipitated confirming interactions between these domains, none of the αA3 mutations affected the efficiency of the MTase-RdRp interaction [182]. In the structure of the full length Japanese encephalitis virus NS5, the MTase domain was found to interact with the backside of the RdRp domain and to only partially cover the top-right corner of the NTP entry channel instead of being located close to the dsRNA channel as previously predicted [181]. Polar/electrostatic interactions as well as a hydrophobic network were observed at the interface between the MTase and RdRp domains. Three conserved RdRp hydrophobic residues, Phe467 at the tip of the ring finger, Phe351 in the βNLS core helix of the index finger, and Pro585 at the tip of the elongated middle finger as well as the MTase residues Pro113 and Leu115 and Trp121 located in a module consisting of residues 112–128 are involved in this interface hydrophobic network. Both Pro113 and Trp121 are highly conserved in flavivirus NS5s. The conserved neighboring GTR sequence (residues 263–265) in the MTase also mediates the MTase-RdRp interaction. The N7 and 2′-*O* methylation activities of the flavivirus MTase domain alone or in the full length NS5 were similar suggesting that the MTase enzymatic functions are not affected by interactions with the RdRp domain [59]. Although in the full length NS5 structure the MTase was located at a distance from the active site of the RdRp, it was postulated that the MTase domain could modulate the movement of the 5-stranded β sheet in the fingers domain during active site closure for RdRp catalysis [181]. The location of the MTase blocks the accessibility of the RdRp amino acids previously predicted to interact with importins and NS3 [173,174]. However, it is still possible that the linker between the MTase and RdRp domains might be able to facilitate the formation of an alternative open NS5 configuration [181]. The results of small-angle X-ray scattering experiments on full length dengue NS5 indicated that this protein was monomeric and well folded in solution but could assume multiple forms ranging from a compact MTase and RdRp domain interacting form to a more extended form with no interaction between the two domains [183]. 

Recombinant NS5 has polymerase activity [184,185,186] as well as uridylyl and adenylyl terminal transferase activities [187]. Both recombinant hepatitis C and bovine viral diarrhea virus RdRps were also reported to have an intrinsic terminal transferase activity [188]. *In vitro* polymerase assays done with recombinant NS5 have been complicated by the terminal nucleotidyl transferase activity which end labels the template. In a recent study, *in vitro* RNA templates were treated with sodium periodate to block the 3'-OH group of the template and this prevented elongation of the template by the NS5 terminal nucleotidyl transferase activity [180]. Templates with a 3' hairpin have also been a problem since instead of *de novo* initiation occurring, the hairpin is used as a primer by the RdRp and a product consisting of the template linked to the newly synthesized extension (2× product) is generated [184,186,189]. However, *de novo* initiation does occur *in vitro* from the 3' end of templates containing the 5' end of a flavivirus genome RNA and produces a complementary minus strand copy the same size as the template (1× product). It has not yet been possible to assemble a functioning multicomponent flavivirus replication complex *in vitro* from recombinant nonstructural proteins. 

## 7. Viral RNA Translation and Replication

Translation of the incoming genome produces the nonstructural proteins needed for viral RNA replication. During the early phase of the virus life cycle, the low number of genomes present switch back and forth between functioning as an mRNA for translation and as a template for minus strand RNA synthesis. The linear form of the genome is used for translation while the cyclized form with the 3'–5' long distance RNA-RNA interactions is required for minus strand RNA initiation [67,68,69,70]. At early times after infection (up to 6 to 8 h), the level of viral RNA synthesis is low, with similar levels of plus and minus strand viral RNAs being produced. Viral minus strand levels remain low throughout the replication cycle. However, once minus strand templates are sequestered within ER membrane invaginations in association with replication complexes and some cellular proteins, the exponential genome synthesis phase begins with multiple nascent RNAs being simultaneously copied from a single template in a semiconservative manner [190]. It was recently hypothesized that 3'–5' basepairing between adjacent genomes could result in the formation of non-covalent concatamers when genome concentrations are high so that individual genomes would not need to switch between the linear (translation) and cyclized (minus strand synthesis) forms [191]. However, such a model would favor efficient minus strand synthesis which is not consistent with the limited amount of minus strand synthesis observed throughout the infection cycle. 

The production of a sufficient amount of viral proteins at early times is required both to counteract cell antiviral defense responses and to remodel the cell in preparation for exponential genome synthesis and virion assembly. Infection of cells with WNV results in the production of Type I interferon. In human cells, WNV infections block phosphorylation of STAT1 and STAT2 [107,192,193], while in mouse cells, phosphorylation of STAT1/2 occurs but nuclear translocation of these molecules is blocked [194]. Viral proteins induce ER membrane proliferation as well as invaginations in the ER membrane that function as sites of exponential genome synthesis [91,195,196]. Host cell cholesterol homeostasis is altered by WNV infection with upregulation of cholesterol biosynthesis and redistribution of cholesterol to ER membrane regions containing active viral replication complexes [197]. 

Differences in the modulation of infected cells by WNV and other members of the genus *Flavivirus* have been reported. WNV infection was shown to activate only the initial stages of the autophagy pathway in cultured cells and neurons [198,199]. While inhibition of autophagy was shown to reduce the replication of dengue 2 virus and Modoc virus [200], neither activation nor inhibition of autophagy affected the efficiency of WNV replication [198,199]. However, activation of autophagy associated PI3 kinases was shown to be important for efficient replication of dengue, Modoc and WNV. Expression of dengue NS4A alone induced autophagy and PI3K activation [200]. Endocytosis of a subset of tight junction membrane proteins, including claudin-1 and JAM-1, followed by lysosomal degradation occurs in epithelial and endothelial cells infected by WNV but not dengue 2 virus [201]. Expression of WNV capsid alone was sufficient for the degradation of tight junction proteins in epithelial cells [202]. Although intracellular cytoplasmic membrane proliferation and rearrangement occurs in cells infected with different types of flaviviruses, the mechanisms involved in activating fatty acid synthesis and also in the lipid microenvironments created differ between flaviviruses. Dengue virus NS3 has been reported to interact with the cellular fatty acid synthetase protein and relocalize it to regions of virus replication [203]. This was not found to be the case for WNV NS3 [204]. Hepatitis C virus and picornavirus infections create specific lipid microenvironments in cells enriched in phosphatidylinositol-4-phosphate that colocalize with viral replication complexes [205]. Efficient WNV replication in infected cells has been shown to require fatty acid synthesis but WNV infections do not redistribute phosphatidylinositol-4-phosphate lipids to their replication complex vesicles [206]

At early times after infection, translation of viral RNA likely occurs by a cap-dependent mechanism. Flavivirus infections do not induce the formation of stress granules which would result in shut down of cap-dependent translation [207,208] and the cellular eIF2α kinase PKR is not activated by viral RNA in infected cells [209]. The low levels of viral RNA present during the initial stages of infection as well as the sequestering of the viral RNA replication complexes in ER membrane invaginations at later stages of infection likely play a role in “hiding” viral RNA from PKR. However, it was also reported that NS2A may block PKR activation [208] and that the C protein of JEV can inhibit stress granule formation through interaction with the cellular protein Caprin-1 [210]. At later times of infection, a large pool of structural proteins must be produced to assemble nascent virions. How flavivirus genome translation efficiently competes with the cell mRNAs, since both have a 5' cap, is not known. The observed upregulation of cap-dependent translation factors prior to 24 h after infection but dephosphorylation of p70S6K and 4E-BP1 at 24 and 48 h [211], as well as the observation of cap-dependent translation of a flavivirus genome when eIF4E was abundant but cap-independent translation when eIF4E levels were low [212], suggests the possibility that the viral RNA may switch to a cap-independent translation mechanism at later times of infection. Both the 3' and 5' UTRs of the flavivirus genome were reported to be required for cap-independent translation. Members of the genus *Flavivirus* do not have an internal ribosome entry site and a specific alternative translation initiation mechanism has not yet been defined. Cell mRNAs form “closed loops” that facilitate translation reinitiation through interactions between 5' cap binding proteins and 3' polyA binding proteins. An internal polyA is present in the tick borne encephalitis virus 3' UTR but deletion of this region or of the entire variable region within the 3' UTR had no effect on genome translation [213]. The 3' UTRs of mosquito borne flaviviruses do not have a poly A but poly A binding protein was reported to bind to an unmapped internal region of the 3' UTR near the dumbbell RNA structures and to facilitate *in vitro* translation [214]. This RNA-protein interaction may facilitate cap-dependent translation at early times of infection. Replicons with mutations/deletions of 3' and 5' dumbbell sequences had reduced translation efficiency suggesting that sequences in the 3' UTR may be involved in regulating translation [55,215]. The cellular far upstream element (FUSE) binding protein 1 was reported to bind to both the JEV 3' and 5' UTRs and to function as a suppressor of viral translation [216]. Although both *in vitro* and *in vivo* translation of JEV RNA was reported to be inhibited by JEV sfRNA [217], the highest levels of sfRNA are present in infected cells when viral protein translation is maximal. 

Expression of high amounts of viral proteins in infected cells can induce ER stress, which activates a signaling network called the unfolded protein response (UPR). At least three mechanistically distinct signaling pathways of the UPR regulate the expression of numerous genes that maintain protein homeostasis in the ER or induce apoptosis if ER stress is not relieved. At late times after infection, differential activation of the UPR pathways was observed in Kunjin infected mouse cells with the ATF6 and IRE1 arms but not the PERK arm being activated and this was postulated to contribute to maintaining cell survival [218,219]. In contrast, in WNV infected human neuroblastoma cells and primary rat hippocampal neurons activation of all three of the UPR pathways as well as of apoptosis pathways was observed [220]. 

## 8. Host Cell Proteins that Interact with the WNV Genomic 3' Terminal SL RNAs and Facilitate RNA Synthesis

A number of cell proteins that bind to regions of the UTRs of flavivirus RNAs have been reported [58,221,222,223,224,225,226,227,228,229,230]. Three cellular proteins with molecular masses of 52, 84 and 105 kDa were reported to bind specifically to probes consisting of the two 3' terminal SLs of the genomes of WNV and 3 other flaviviruses [231]. The 52 kDa protein was identified as eukaryotic elongation factor 1 alpha (eEF1A) and one major (60% of the binding activity) and two minor (each ~20% of the binding activity) binding sites for eEF1A on the WNV genome RNA 3' SL were mapped by RNase footprinting and filter-binding assays (indicated at stars in Figure 1B) [221]. The binding activity of eEF1A for the 3' terminal RNAs of four divergent flaviviruses was similar and strongly suggested that this interaction is a common characteristic of the genomes of the members of the genus *Flavivirus* [47]. eEF1A is the second most abundant protein in cells after actin and its primary cellular function is to ferry aminoacylated tRNAs to ribosomes [232]. Base substitutions were engineered in the major or minor eEF1A binding sites in a WNV infectious clone and their effects on plus and minus strand viral RNA synthesis in RNA transfected cells were analyzed. Three of the four nts of the major binding site are typically basepaired in the 3' terminal SL. Mutation of these nts decreased *in vitro* eEF1A binding while disruption of the base pairs by substitution of the pairing partners increased *in vitro* eEF1A binding [221]. Mutations that decreased *in vitro* eEF1A binding to the 3' SL RNA also decreased minus strand levels in transfected cells and mutations that increased *in vitro* eEF1A binding increased minus strand RNA levels [233]. Interestingly, increased synthesis of minus strand viral RNA reduced the amount of progeny genome produced, indicating that the correct ratio of minus to plus strand RNA is important for the overall virus life cycle. Even though the two minor eEF1A binding sites each accounted for only ~20% of the *in vitro* eEF1A binding activity for the 3' SLs, mutation of some of the nts in the minor eEF1A binding site located in the top loop of the 3' terminal SL or of the other minor site located in the sHP, either by deletion of the sHP, by mutation of G87 to C, or deletion of G87 in the loop of the sHP, was lethal for a WNV infectious clone [47]. A 53% decrease in *in vitro* eEF1A binding activity was observed with a WNV 3' SL probe that had a G87C substitution indicating that G87 is important for efficient eEF1A binding to the viral 3' SL RNA. Interestingly, substitution of this nt with a C had a greater negative effect on *in vitro* eEF1A binding activity than deletion of the entire sHP. Substitution of G87 with an A or U reduced eEF1A binding by 42% and 34%, respectively. Consistent with the different eEF1A binding activities, the G87C mutation was lethal in an infectious clone but viral RNAs with either an A or a U at this position were still able to carry out a sufficient amount of viral RNA synthesis to generate a revertant with the parental G. 

Even though the nts involved in flavivirus genome 3'–5' basepairing have been identified, little is known about the mechanisms regulating the formation and disruption of this long distance RNA-RNA interaction. However, the finding that eEF1A binds to three sites on the genome 3' RNA structures and that the interaction between eEF1A and the WNV 3' SLs facilitates minus strand RNA synthesis strongly suggests that eEF1A is involved in mediating the transition from basepairing between nts located in the bottom part of the 3' terminal SL to 3'–5' RNA-RNA basepairing. A *cis*-acting metastable structural feature located in the middle of the terminal 3' SL that consists of small, symmetrical bulges flanking two base pairs was identified [47]. The high conservation of both this structural feature and of the nts of the two basepairs in the genome RNAs of divergent flaviviruses as well as of the location of this feature at the top of the region in the 3' terminal SL that unpairs to form the long distance 3'–5' RNA-RNA interaction suggest that this structural feature is also involved in regulating the switch from a 3' SL RNA-RNA interaction to a 3'–5' RNA-RNA interaction.

It is expected that initiation of plus strand synthesis would occur from the minus strand product. However, how the 3' end of a nascent minus strand is released from the duplex formed with the genome template so that it can initiate plus strand synthesis is not known (Figure 2C). The copying of a nascent genome from the new minus strand would release the original genome template. During the early stage of the virus replication cycle, the reinitiation of genome synthesis is not efficient and similar low levels of plus and minus RNA are made (Figure 2D). The binding of the NS5 MTase to the 5' terminal nts of a nascent plus strand product and then to sites on the 5' SLA as it forms in the nascent plus stand as part of the cap addition process may facilitate release of the 3' end of the minus strand and formation of the terminal 3'SL on the minus strand template needed for reinitiation of plus stand synthesis. 

Four cellular proteins, p108, p60, p50, and p42, were reported to bind specifically to the 3' terminal SL of the WNV minus strand RNA [58]. The 42 kDa protein was identified as T cell-restricted intracellular antigen-1 (TIA)-related protein (TIAR) and the closely related protein, TIA-1 was also shown to bind to the 3' SL of the minus strand RNA [234]. These multifunctional proteins are members of the RRM family of RNA-binding proteins, bind to AU rich RNA sequences, are expressed in most tissues and shuttle between the nucleus and cytoplasm of infected cells [235,236,237,238]. The binding sites for TIAR and TIA-1 were mapped to two 5 nt AU sequences located in the two side loops on the 3' terminal SL of the WNV minus strand [239]. The AU sequences of both loops are required for efficient *in vitro* binding of TIAR and TIA-1 and recent data suggest that one TIAR protein binds to each loop and that protein binding occurs in a cooperative manner [240]. Deletion or C substitution of the AU-rich sequence in either loop in a WNV infectious clone was lethal and partial deletion or substitution of these sequences reduced the efficiency of virus replication [239]. The degree of decrease in efficiency of plus strand RNA synthesis and virus production correlated with the degree of decrease in efficiency of *in vitro* protein binding, but minus strand RNA levels were similar for the different mutant RNAs suggesting that the interaction between TIA-1/TIAR and the 3' terminal SL of the WNV minus strand RNA facilitates exponential genome RNA synthesis from the minus-strand template. These mutations did not affect the translation efficiency of the genome RNA. The observation that the replication of WNV, but not that of viruses from other families, was less efficient in TIAR-knockout cell lines than in wild type cells also suggested a functional role for these proteins during WNV replication [234]. 

## 9. Nonstructural Protein Interactions and Regulation of Viral RNA Replication

In a recent study, individual WNV nonstructural proteins were expressed in Vero cells in different combinations and their interactions were analyzed by various imaging techniques, including confocal microscopy, fluorescence resonance energy transfer, and biologic fluorescence complementation [153]. NS5 was found to only interact with NS3. Previous studies done with dengue NS3 and NS5 proteins also demonstrated interaction between these two proteins and suggested that the phosphorylation state of NS5 affected the interaction [241]. The interacting regions of the dengue proteins were mapped to the *C*-terminus of NS3 (residues 303 to 618) and the *N*-terminus of NS5 (residues 320 to 368) with (Lys-330) identified as the NS3 helicase domain interacting residue [179,241]. Both the *in vitro* NTPase and RTPase activities of dengue NS3 were enhanced by addition of recombinant dengue NS5 [242,243] suggesting the possibility that regulation and coordination of the NS3 NTPase, RTPase and helicase activities and the NS5 polymerase and capping activities during viral RNA synthesis may be mediated by interactions between NS3 and NS5. Although similar *in vitro* studies have not yet been done with WNV NS3 and NS5 proteins, it is speculated that the results would be similar to those found with the dengue proteins. 

In the same recent study, consistent with previous data, NS3 was found to also interact with its membrane associated co-factor NS2B [153]. NS2B was also found to interact with the three other membrane-associated proteins, NS2A, NS4A and NS4B, and therefore appears to be the critical protein for bringing these four proteins together in a complex (Figure 2). Although an NS2A-NS4A interaction was observed, neither NS2A-NS4B nor NS4A-NS4B interactions were detected. No interactions between NS1 and other nonstructural proteins were detected in this study. Dengue NS1 was previously found not to trans-complement a yellow fever virus genome with an NS1 deletion but genome variants were selected that could utilize the dengue NS1 and sequencing detected a single point mutation in the NS4A gene suggesting that an interaction between NS1 and NS4A is required for early viral RNA synthesis [244]. In contrast, a recent study obtained both genetic and physical evidence of interaction between WNV NS4B and NS1 [245]. In this study, a WNV genome encoding a mutant NS1 (RQ10NK) that showed enhanced NS1 secretion but impaired virus replication was used to identify suppressor mutations. A compensatory mutation in the viral NS4B gene rescued the impaired replication phenotype of the NS1 mutant but had no effect on the replication of the wildtype genome. Additional studies, including immunoprecipitation and NMR experiments, detected a physical interaction between NS1 and NS4B. 

At early times of infection when the number of genomes present is low, genomes are expected to switch back and forth between the cyclized replication form and the non-cyclized translation form. How this switching is regulated is not known. Natural lineage 1 and lineage 2 WNV strains produce low levels of viral RNA at early times after infection but produce a sufficient amount of viral protein to block the Type I IFN response [194,206]. These findings suggest that translation is favored over minus strand synthesis at early times of infection. In contrast, the W956IC lineage 2/1 chimeric infectious clone virus produces higher levels of early viral RNA and the higher early viral RNA levels activate PKR leading to efficient induction of SGs [206,208]. Lower early viral protein levels and less efficient counteraction of the Type 1 IFN response were also observed in W956IC infected cells suggesting that cyclized genomes predominated at early times [246]. The majority of the W956IC sequence is from 956D117B3 (lineage 2) but the 3' 1,496 nts (NS5 *C*-terminal 287 amino acids and the 3' UTR) are from Eg101 (lineage 1) [247]. The phenotype of the W956IC chimera suggested that interactions between specific residues of the viral nonstructural proteins may be involved in down-regulating the efficiency of early viral RNA synthesis. The low early viral RNA level phenotype of the natural virus strains was rescued by replacement of the combination of the *C*-terminal 23 aa of E plus NS1 plus the *C*-terminal 439 aa of NS3 plus the *N*-terminal 105 aa of NS4A with Eg101 sequence in a clone with the chimeric NS5 but not by replacement of any of these individual regions. Rescue by this combination of sequences suggests the possibility of functionally important interactions between the *C*-terminal regions of NS3 and NS5 as well as between NS1 and NS4A. Suppression of early viral RNA levels was also observed when the *C*-terminal 214 aa of NS4B and the full length NS5 from Eg101 were inserted. The addition of the Eg101 NS1 region to the latter clone did not further decrease early RNA replication. Rescue by this combination of sequences suggested that interactions between the NS5 *N*- and *C*-terminal regions and possibly also between NS5 and NS4B are important. Interestingly, viral replication was less efficient for chimeras with a hybrid NS5 than for ones with full length Eg101 NS5. The majority of the residues that differ between the 956D117B3 and Eg101 NS5 sequences are predicted to be on the protein surface and none appear to be directly involved in RdRp function supporting the hypothesis that some of these residues may be protein interaction sites. Substitutions at R325, R519-K523, R769 and K840-R841 in a dengue NS5 had no effect on *in vitro* polymerase activity but were lethal in an infectious clone [180]. Also, dengue NS5 mutants that had wildtype *in vitro* polymerase activity as recombinant proteins caused impaired virus replication when they were present in an infectious clone [180]. The finding that substitution of single residues in the WNV RdRp located near the rNTP binding pocket but not in direct contact with the incoming rNTP altered both plaque morphology and the kinetics of viral RNA replication also suggest subtle changes in the RdRp that are remote from the active site are able to affect function. The biological fitness of these mutants was tested in a number of insect cell lines and Vero cells as well as in one day old chickens and *Culex pipiens* mosquitoes. While all of these mutants were significantly attenuated for virus growth in the cell lines and chickens with the NS5 A365N mutant having the greatest negative effect, all of the mutants grew as efficiently as wildtype virus in *Culex* mosquitoes indicating that the effects on virus fitness were host dependent [248]. The data from all of the above studies suggest that interactions between NS5 and other viral nonstructural proteins as well as cellular proteins regulate the RNA synthesis functions of NS5 and this regulation may differ at early and late times after infection and in different host species. 

Dengue and WNV NS5 proteins are phosphorylated on serine and threonine residues by cellular serine/threonine kinases and it was suggested that the phosphorylation state of NS5 may regulate the association between NS5 and NS3 [241,249,250]. However, these and other modifications could also regulate interactions between these viral proteins and cell proteins. A number of cell proteins have been reported to interact with flavivirus NS5 or NS3. WNV NY99 NS5 was shown to inhibit STAT1 phosphorylation in human cells but only when it was expressed from a polyprotein [251]. NS5 was reported to interact with eIF3L [252] and Hdj2 (a heat shock protein 40/DnaJ homolog) [253]. The *C*-terminal regions of both tick borne encephalitis NS5 and WNV NS5 were predicted to contain four *C*-terminal PAZ binding motifs but only the TBEV NS5 had two additional PAZ motifs in the MTase region that were shown to modulate viral replicon replication [254]. The *C*-terminal PAZ motifs of the TBEV and WNV NS5s were found to interact with different subsets of host PAZ proteins. In another study, a high-throughput yeast two-hybrid screen identified 108 human proteins interacting with either NS3 or NS5 or with both proteins [255]. The functional relevance of the majority of the cellular proteins reported to interact with NS5 has not yet been tested. However, in some cases, even though a robust *in vitro* interaction was demonstrated, the functional relevance of the interaction in infected cells could not be demonstrated [252]. It is possible that the surfaces of NS5 that are exposed when it is expressed alone as a recombinant protein differ from when it is in the context of the complete replication complex in an infected cell.

## 10. Progeny Virus Assembly and Release

During the exponential genome synthesis phase, nascent genomes exit replication complex vesicles through a pore [196]. A recent study suggested that the nascent flaviviral RNAs may exit into a specialized compartment in the cytosol that provides a protective environment [256]. Nascent genomes can be replicated, translated or assembled into a virion in association with ER membranes. It is thought that the hydrophobic face of C dimers binds to the cytoplasmic side of the ER membrane while the charged face binds to genomic RNA in a sequence nonspecific manner [10,257]. This idea is consistent with the observation that large deletions in the central hydrophobic region of the C protein of TBEV were tolerated [258] and that no specific encapsidation signal sequence in flavivirus genomic RNA that was recognized by the C protein could be identified [259]. The transmembrane domains of the E and prM proteins insert into the ER membrane with their exodomains located in the ER lumen. The assembly of virions is thought to occur due to interaction of a genomic RNA with membrane-associated capsid dimers in areas where E and preM proteins are also located leading to immature virion budding into the ER lumen. Recent data also suggest that NS2A may play a role in virion assembly [101]. prM and E heterodimers form trimer spikes on the surface of the immature virions. In this conformation, the E fusion peptide is protected from triggering premature fusion with cellular membranes in the mildly acidic compartments of the cell secretory pathway during virion egress [260,261]. The immature virions are transported through the Golgi and secretory pathway where glycans on E and prM are modified. After the *N*-terminal portion of prM is cleaved in the trans-Golgi compartment by a cellular furin-like protease [262], the 60 E protein trimers undergo conformational change, rotation and rearrangement to form 90 antiparallel dimers that create a “smooth” protein shell with icosahedral symmetry on the outer side of the viral envelop [262,263,264,265]. Mature virions are transported to the plasma membrane in vesicles and released by exocytosis. Typically, WN virions are released from infected cells starting at 8 to 10 h after infection and peak extracellular virus titers are usually observed by 24 h. Smooth, noninfectious, subviral particles called slowly sedimenting hemagglutinin (SHA), that are composed of a circular cellular membrane inserted with E and M proteins as well as some prM are also produced by infected cells [266,267].

The specific involvement of a number of cell proteins in the assembly, transport and release of flaviviruses has been suggested. Interaction of WNV C protein with the nucleolar helicase DDX56 was reported to enhance the assembly of infectious virions [268] and a subsequent study showed that the helicase activity of DDX56 was required for its role in assembling infectious virions [269]. Studies with a WNV chimera lacking the C coding region and expressing dengue 2 prM and E instead of the WNV surface proteins, showed that mutations in NS2A or NS3 independently enhanced genome packaging and also that genomes that did not express NS1' were packaged more efficiently [147]. Src family kinase c-Yes activity was reported to be required for transit of assembled immature WN virions from the ER through the secretory pathway [270]. Alix, a cellular protein associated with the ESCRT machinery, was reported to interact with yellow fever virus NS3 and expression of dominant negative Alix proteins inhibited virion release [271]. Increased accumulation of intracellular infectious virions was observed in dengue virus infected cells deficient in AP-1, a heterotetrameric adaptor protein complex involved in trafficking cargo molecules in the biosynthetic pathway back and forth between the trans-Golgi network and endosomes [272]. A conserved capsid region sequence in the dengue genome RNA was also reported to enhance infectious virion assembly [273] but it is not known what proteins interact with this RNA region.

## 11. Conclusions

Although much has already been learned about the replication and molecular biology of flaviviruses, there are still many unanswered questions. Data from multiple studies confirm that flaviviruses utilize cell proteins during each step of their replication cycles but the roles of most of these host factors in virus replication are still not well understood. WNV infection alternates between insect vectors and vertebrates in nature and some of the host factor proteins used by flaviviruses may differ in mammalian and insect hosts. Interactions between viral nonstructural proteins and also between these proteins and cellular proteins are likely to be complex and may occur only after expression of the viral proteins from the viral polyprotein and in the context of an infected cell. Also, infections initiated by genomic RNA transfection instead of virus infection would not activate the cell signaling pathways typically activated by virus attachment and entry. Activation of cell signaling pathways at early times have been shown to have sustained effects throughout the infection cycle. Activation of cell signaling pathways may also be required for appropriate post-translational modification of viral proteins needed to regulate their functions. Although the 3' and 5' genome sequences involved in the long distance RNA-RNA interaction have been identified, the mechanisms regulating switching between the linear and cyclized forms of the genome are not known. Although a number of interesting insights have been obtained about the early stages of the flavivirus replication cycle and about the mechanisms used by different flaviviruses to remodel infected cells, there is still much that is not known. 
